# Evaluation of four scoring systems in prognostication of acute pancreatitis for elderly patients

**DOI:** 10.1186/s12876-020-01318-8

**Published:** 2020-06-01

**Authors:** Yajie Li, Jun Zhang, Jihong Zou

**Affiliations:** Geriatrics Department, Zhongda Hospital Southeast University, No.87, Dingjiaqiao, Nanjing, China

**Keywords:** Acute pancreatitis, Elderly patients, Prediction, Scoring system, ROC (receiver operating characteristic) curve

## Abstract

**Background:**

To evaluate the ability of four scoring systems (Ranson, BISAP, Glasgow, and APACHE II) to predict outcomes of acute pancreatitis (AP) in elderly patients.

**Methods:**

This was a retrospective study of 918 patients presenting with AP at Zhongda Hospital Southeast University, from January 2015 to December 2018. We divided patients into two groups: 368 patients who were ≥ 60 years old, and 550 patients who were < 60 years old. Four scoring systems were used to analyze all patients.

**Results:**

The severity of the disease, and mortality were significantly different between the two groups (*p* < 0.05), while the difference between the two groups about pancreatic necrosis is statistically insignificant (*p* = 0.399). The differences of the AUCs (Area under curves) for prediction of outcome of SAP (severe acute pancreatitis) between the two groups were statistically significant for Ranson and APACHE II (*p* < 0.05), but not for the differences between BISAP and Glasgow. All the four scoring systems were similar in terms of prediction of pancreatic necrosis and death in both groups.

**Conclusions:**

Prediction of severity, pancreatic necrosis, and death in AP for elderly patients can be performed very well by using BISAP. APACHE II is more suitable for younger patients when dealing with severity. Ranson and Glasgow can be used to evaluate all AP patients in most cases; however, Ranson is more effective for younger patients when used to assess severity.

## Background

Acute pancreatitis (AP) is one of the most common gastrointestinal conditions that causes hospitalization [1, 2]. Over the past decade, there has been a large increase in admissions worldwide [3–5]. Many countries such as the United States, Japan, and China are now facing accelerating population aging [6]. By 2030 in China, according to the National Health Commission of the People’s Republic of China, the population older than 60 years of age will exceed 30% of the total population. This suggests that the total number and the proportion of elderly patients among acute pancreatitis patients will increase significantly in the future.

About 80% of the acute pancreatitis cases are mild and self-limited with no sequelae. The remaining cases deteriorate, and necrosis arises in parts of the pancreas and surrounding tissues. Despite the fact that mortality associated with acute pancreatitis has continuously reduced [7], the overall mortality of AP is 2–8% [8, 9]; however, when the cases become severe, mortality can reach to about 20–30% [10]. The elderly patients are a subgroup at particularly higher risk in terms of mortality form AP [11]. In recent research, the mortality rate is 9-fold higher in patients older than 59 years than in those younger than 59 years [12]. Elderly patients have more comorbidities, that increases their mortality further [13]. Because of the higher mortality of severe acute pancreatitis (SAP) and the causes of death related to elderly patients, it is necessary to perform careful ongoing clinical evaluations, the results of routine laboratory and radiographic testing, together with multi-factorial scoring systems to predict SAP [14].

Several scoring systems are available, including the Ranson criteria [15], which was the first AP scoring system that can be used to evaluate biliary and non-biliary pancreatitis. The Glasgow scoring system [16] is similar to the Ranson criteria, and it is also based on objective clinical indicators; the assessment needs to be completed within 48 h after admission. The Acute Physiology and Chronic Health Evaluation (APACHE) II [17] was originally developed for critical patients in ICU (intensive care unit), and was first used for evaluation of AP in 1989. A simple evaluation method named the bedside index of severity in acute pancreatitis (BISAP), was proposed in 2008 [18]. BISAP can be used to estimate the severity of AP in the early phase. All these scoring systems can be applied together with the ongoing evaluation by the clinician to provide more accurate and rapid diagnosis.

Many studies have evaluated the accuracy of these scoring systems for estimating the severity of acute pancreatitis [19, 20], nevertheless, few have validated these systems in elderly AP patients. Therefore, the aim of this paper was to evaluate the effectiveness of these four aforementioned scoring systems in the prediction of severity, pancreatic necrosis, and death from acute pancreatitis in elderly patients.

## Methods

### Study design

A retrospective analysis was carried out. The patients were selected complied with following requirements:

Inclusion criteria: Primary diagnosis compliant with acute pancreatitis .

Exclusion criteria: (1) incomplete data; (2) diagnosis of AP was in doubt; (3) presence of other serious diseases (including chronic pancreatitis, chronic cardiac failure: New York Heart Association level IV, chronic obstructive pulmonary disease, chronic renal insufficiency requiring long-term maintenance hemodialysis, cirrhosis, and tumor);and (4) death within 48 h of admission.

Finally, we retrospectively identified 918 adult patients with a diagnoses of acute pancreatitis treated at Zhongda Hospital Southeast University (Nanjing,China), from January 2015 to December 2018 .

The patients were further divided into two groups: Aged group (≥60 years old), and younger group (< 60 years). For both groups, four scoring systems were used: Ranson criteria, Glasgow, APACHE-II, and BISAP. The BISAP, APACHE II scores were assessed according to the data of the patient’s admission within 24 h, and the Ranson and Glascow scores were scored at the admission and within 48 h. All scores were calculated for the most severe laboratory tests and vital signs during the evaluation period (time required by the scoring system to observe). The AUCs of the various scoring systems for predicting severity, pancreatic necrosis, and mortality were obtained from their ROC curves. For each scoring system, the statistical differences of AUCs between the two groups were analyzed.

### Diagnostic criteria

The diagnostic criteria for acute pancreatitis is determined in accordance with the 2012 revision of the Atlanta classification [21]. The patient should have at least two of the following three diagnostic features:

1. Consistent abdominal pain with acute pancreatitis.
2. Serum amylase and/or lipase levels that are at least 3 times higher than the upper limit of the normal range.
3. Findings of acute pancreatitis on computed tomography (CT) or magnetic resonance imaging (MRI).

According to revised Atlanta classification [21], the absence of organ failure and local or systemic complications is characterized as mild acute pancreatitis (MAP). The presence of local or systemic complications or transient (less than 48 h) organ failure is characterized as moderately severe acute pancreatitis (MSAP). Persistent (longer than 48 h) organ failure (may be single or multiple organ failure) is characterized as severe acute pancreatitis (SAP).

Organ failure included one or more of the following

1. Shock/cardiovascular failure: Systolic blood pressure less than 90 mmHg or basal systolic arterial pressure reduced more than 40 mmHg, accompanied with signs of tissue hypoperfusion (lactate larger than 3 mmol/L); saturation of central venous oxygen (SvcO2) less than 70%.
2. Pulmonary insufficiency: Arterial PO2 less than 60 mmHg in room air or mechanical ventilation required.
3. Acute renal failure: Serum creatinine level > 2 mg/dl after hemodialysis or rehydration indicating a score no less than 2 according to modified Marshall scoring system.

After the first week of the disease, CECT showing non-enhancement of pancreatic parenchyma was defined as pancreatic necrosis.

### Treatment

According to “Guidelines for the diagnosis and treatment of acute pancreatitis in China (2013)”, the following treatments were given to patients diagnosed with AP:

Nil per oral, early fluid resuscitation (in the first 24 h, the fluid resuscitation dose should be 5–10 ml·kg^− 1^·h^− 1^), nutritional support (for hemodynamic stability, with enteral nutrition started within 24–48 h if possible), pain control, application of proton pump inhibitor, antibiotic,and somatostatin or its analogues. Organ functional support was given to patients with organ dysfunction (i.e., mechanical ventilation, continuous renal replacement therapy, or treatment with vasoactive drugs).

### Statistical analysis

SPSS version 20.0 (IBM Corp.) was used for statistical calculations: Receiver-operating curve (ROC) was used for assessing the prognostic value of each scoring system, the area under the curve (AUC) of the four scoring systems were calculated individually for both groups, and the AUCs of the same system were compared with one another. *P*-values < 0.05 indicates statistically significant.

## Results

A total of 918 patients with AP (age range 21–89 years, mean age 58.4 ± 18.1) were hospitalized. They were divided into two groups (Table 1): The aged group (aged 60–89 years, mean age 73.83 ± 7.78) and the younger group (aged 21–59 years, mean age 42.10 ± 9.50). Of the 368 elderly patients, 27 (7.3%) developed severe acute pancreatitis, 28 (7.6%) developed pancreatic necrosis and 11 (3%) died. In the control group, among 550 younger patients, 25 (4.5%) developed severe acute pancreatitis, 34 (6.2%) developed pancreatic necrosis and 5 (0.9%) died. For both groups,there were more males than females. The male/female ratios were 201:167 and 359:191 in the aged and younger groups,respectively.

**Table 1:** Comparisons of the two groups

Variables		Elderly patients	Younger patients	*χ*^2^	P-value (*p* < 0.05)
Male:Female		201:167	359:191	–	–
Mean age (years)		73.83 ± 7.78	42.10 ± 9.50	–	–
Etiology	Gall Stone	276 (75.0%)	283 (51.5%)	–	–
	Alcoholic	21 (5.7%)	116 (21.1%)		
	hyperlipemia	26 (7.1%)	83 (15.1%)		
	others	45 (12.2%)	68 (12.3)		
Comorbi-dities	DM^*^	76 (21.2%)	126 (22.9%)	0.654	0.419
	CHD^**^	36 (9.8%)	11 (2%)	27.491	< 0.01
Severity	MAP	316 (85.9%)	506 (92%)	8.849	< 0.01
	MSAP	25 (6.8%)	19 (3.5%)	5.386	< 0.05
	SAP	27 (7.3%)	25 (4.5%)	3.871	< 0.05
Organ failure	transient	6 (1.6%)	12 (7.3%)	0.349	0.555
	persistent	27 (7.3%)	25 (4.5%)	3.871	< 0.05
Pancreatic necrosis		28 (7.6%)	34 (6.2%)	0.713	0.399
Death		11 (3%)	5 (0.9%)	5.570	< 0.05

* = Diabetes Mellitus, ** = coronary heart disease

The clinical characteristics of the two groups are displayed in Table 1. The proportion of severity, persistent organ failure, pancreatic necrosis, and mortality were all higher in the aged group than in the younger group. Statistically significance was be observed between the two groups with respect to the differences of severity, persistent organ failure, and mortality among AP patients, while the difference of transient organ failure and pancreatic necrosis between elderly and younger AP patients was insignificant.

The AUCs of the four scoring systems for predicting severity of AP were obtained from their ROC curves and are displayed Table 2. For the aged group, BISAP had the largest AUC of 0.922 (95% CI, 0.890–0.947) for prediction of the severity, and was significantly higher than that of APACHE II 0.784 (95% CI, 0.729–0.817, *p* < 0.05). The AUCs for Ranson and Glasgow were 0.867 (95% CI, 0.828–0.900), and 0.913 (95% CI, 0.880–0.940) respectively. For the younger group, for prediction of severity, Ranson had the largest AUC of 0.964 (95% CI, 0.945–0.978), while the AUC of BISAP was 0.942 (95% CI, 0.881–0.969), very similar to that of APACHE II 0.951 (95% CI, 0.884–0.975, *p* > 0.05), and was slightly higher than that of Glasgow 0.881 (95% CI, 0.851–0.907, p > 0.05). Cutoffs were calculated based on the highest sensitivity and specificity achieved from ROC curves [20]. For the aged group, the cutoffs for the four scoring systems were Ranson ≥4 (sensitivity 0.814, specificity 0.842), BISAP ≥3 (sensitivity 0.889, specificity 0.865), APACHE II ≥9 (sensitivity 0.852, specificity 0.610), and Glasgow ≥3 (sensitivity 0.852, specificity 0.842), For the younger group, the cutoffs for the four scoring systems were Ranson ≥3 (sensitivity 0.920, specificity 0.928), BISAP ≥2 (sensitivity 0.960, specificity 0.880), APACHE II ≥8 (sensitivity 0.960, 9specificity 0.930), and Glasgow ≥2 (sensitivity 0.800, specificity 0.882). Using these cutoffs, the sensitivity, specificity, PPV, and NPV were calculated.

**Table 2:** Values of the four scoring systems in prediction of SAP, and comparisons of ROC curves between two groups

Scoring system	AUC	95%CI	Cut-offs	Sensitivity	Specificity	Youden Index	PPV	NPV	Significa-nce level
aged group/ younger group									
Ranson	0.867/0.964	0.828–0.900/0.945–0.978	≥4/≥3	0.814/0.920	0.842/0.928	0.613/0.809	0.289/0.377	0.983/0.996	< 0.05
BISAP	0.922/0.942	0.890–0.947/0.881–0.969	≥3/≥2	0.889/0.960	0.865/0.880	0.754/0.764	0.343/0.276	0.990/0.998	0.383
APACHE II	0.784/0.951	0.729–0.817/0.884–0.975	≥9/≥8	0.852/0.960	0.610/0.930	0.462/0.899	0.147/0.429	0.981/0.998	< 0.01
Glasgow	0.913/0.881	0.880–0.940/0.851–0.907	≥3/≥2	0.852/0.800	0.842/0.882	0.656/0.650	0.299/0.244	0.986/0.989	0.506

**Fig. 1:**
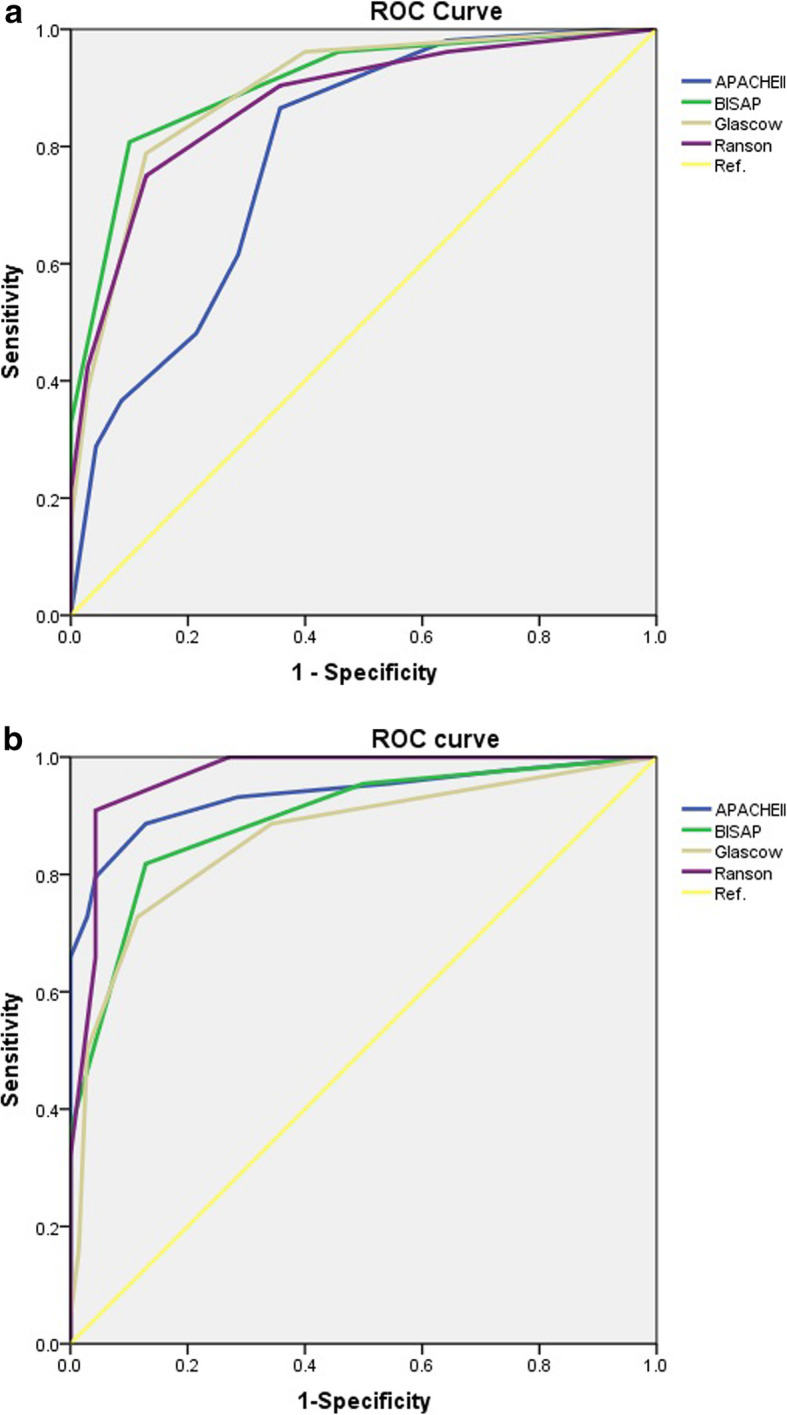
ROC curves for four scoring systems in evaluation of severity (a) aged group (b) younger group

The comparisons of the four scoring systems for prediction of the severity in AP between the two groups are displayed in Table 2. For both groups, BISAP and Glasgow had similar effectiveness (*p* = 0.383 and *p* = 0.506). By contrast, the accuracy of Ranson and APACHE II for prediction of severity for the younger group was significantly higher than for the aged group (*p* < 0.05 and *p* < 0.01). The ROC curves for the four scoring systems for prediction of severity of AP among elderly and younger patients are shown in Figs.1(a) and (b), respectively.

The comparisons of the four scoring systems for prediction of pancreatic necrosis are displayed in Table 3. The AUC of Ranson for the aged group was insignificantly greater than for the younger group (*p* = 0.105), while other three systems had larger AUCs for the younger group. The differences for evaluation of pancreatic necrosis between the two groups according to the four systems were insignificant. All the four scoring systems were more effective for prediction of death in the younger group (Table 4); however, the differences between the groups were insignificant .

**Table 3:** Comparisons of ROC curves for four scoring systems in evaluation of pancreatic necrosis between the two groups

Scoring system	Pancreatic necrosis (AUC)		Significance level
	Aged Group	Younger Group	
Ranson	0.931	0.866	0.105
BISAP	0.824	0.893	0.180
APACHE II	0.855	0.937	0.083
Glasgow	0.853	0.874	0.697

**Table 4:** Comparisons of ROC curves for four scoring systems in evaluation of death between the two groups

Scoring system	Death (AUC)		Significance level
	Aged Group	Younger Group	
Ranson	0.870	0.944	0.138
BISAP	0.891	0.919	0.625
APACHE II	0.918	0.919	0.986
Glasgow	0.899	0.951	0.258

## Discussion

We divided 918 AP patients into two groups: the aged group and the younger group. As summarized in Table 1, differences between the groups of AP patients with diabetes were statistically insignificant, similar to findings from a study in China [22], This suggests that diabetes may be not relevant to the age factor in AP patients. There was a statistical difference between the two groups of AP patients with coronary heart disease, because the incidence of coronary heart disease increases with age. Significantly higher risk of severe pancreatitis (MSAP and SAP), persistent organ failure, and death was also be found in the aged group. This is probably because the functions of various organs decreases with age [23]; therefore, elderly patients are more likely to suffer organ failure. In addition to severity and older age [11], nosocomial infections can also increase the mortality of AP patients [24]. Unfortunately, elderly patients are susceptible to infection, which further increasing the risk of mortality. Frequently use of antibiotic (AB) in Eastern European and Asian countries is also related to increased incidence of nosocomial infections [25], and this makes the situation for elderly AP patients in China even worse. The rate of pancreatic necrosis was similar for both groups in our study; however, highest mortality was related to infected pancreatic necrosis [26], with mortality reaching as high as 30% [11]. These findings suggest that special attention and treatment are necessary for elderly patients.

Among the four scoring systems, age contributed to the scores (Ranson: + 1 point for age > 55; Glasgow: + 1 point for age > 55; APACHE II: + 1 point for age between 45 and 54, + 2 points for age between 55 and 64, + 3 points for age between 65 and 74, + 4 points for age ≥ 75; BISAP: + 1 point for age > 60;). According to Tables 2-4, for prediction of severity, Ranson for elderly patients was less useful than it was for younger patients. Nevertheless, the Ranson scoring system was equally effective when applied to evaluation of pancreatic necrosis and death for both groups. Among the four systems, Ranson showed the best performances for prediction of pancreatic necrosis in elderly patients. When using Ranson to predict SAP for the aged group, the score should be ≥4, similar to findings in [27]. For the younger group, the score is ≥3, which is the same as the criterion in [28].

The Glasgow score is calculated based on objective clinical indicators. The evaluation is mostly used in Europe [19]. The results from our hospital suggest that there was similarly good predictive ability for severity, pancreatic necrosis, and death for both groups of AP patients. The predictive ability of Glasgow was similar to that of the Ranson score [27]. In [28], Glasgow ≥3 was used to diagnose SAP. In the present study, Glasgow ≥3 and Glasgow ≥2 were the criteria for predicting SAP among the AP patients in the aged and younger groups, respectively.

Through many years of practice, APACHE II has been the most widely used AP scoring system, and it is recommended in a number of guidelines [29, 30] . According to our study and that of [31], APACHE II is the most accurate in prediction of mortality for elderly patients. However, this scoring system is complex and cumbersome [11]; and according to the present study, it was not as effective for prediction of severity in the elderly as for younger AP patients. For APACHE II, the cut-off ≥8 is generally accepted as the criterion for diagnosis of SAP [28]. While in [32, 33], APACHE II ≥6 and APACHE II ≥5 were used as cut-offs, respectively. According to this study, to assess severe AP patients, APACHE II ≥9 is needed for the aged group, and APACHE II ≥8 is needed for the younger group.

BISAP is a simple scoring system. The required data can easily be obtained at the time of admission. The in-hospital death rate can be predicted in early stages of AP [34]. Organ failure can be predicted accurately by using this scoring system [10]. In the present study, for prediction of severity, pancreatic necrosis and death, BISAP was useful for both elderly and younger patients. For elderly patients, BISAP showed the best ability in terms of prediction of severity. In some studies, when BISAP ≥2, the patient should be treated as having SAP [33, 35]. In other studies, BISAP ≥3 was used to determine patients with SAP [36–39]. In the present study, the criterion for predict SAP was different for the two groups: (aged group: BISAP ≥3, younger group: BISAP ≥2).

For the aged group, even if the condition is mild, the score is still likely to be higher than that of the younger group. In the present study, the scoring cutoffs for the aged group were one point higher than for the younger group. These changes can increase the specificity while slightly reducing the sensitivity of the four scoring systems. CTSI and MTSI are good predictors of both mortality and AP severity [27, 40]. Although their calculation require radiological expertise, they provide more precise information if they are combined with the four aforementioned scoring systems in the future.

## Conclusions

Elderly AP patients are more susceptible to severe disease, organ failure and death while in hospital. More attention, appropriate triage, and early prevention should be provided for these patients. For the prediction of severity, BISAP is the most appropriate scoring system. Ranson and APACHE II for elderly patients are not as useful as they are for younger patients. All four scoring systems show similar performances with respect to prediction of pancreatic necrosis and mortality between elderly and younger patients. Either score of the four scoring systems can be used to determine whether the risks of SAP are different between two groups. The results suggest that we should distinguish between younger and the elderly patients when using these scoring systems to determine whether they are at risk of SAP. In the present study, we used 60 years as the cutoff age [41]. Change in age selection has an impact on grouping and may affect the final outcome. Therefore, for some areas with different age distributions from those of China (for example: where people tends to live longer), changes in the cutoff age and further research are necessary.

## Data Availability

The [SPSS data file] data used to support the findings of this study are available from the first author (Ms. Li Yajie) upon request. Anyone need the data can contact the following address: Ms. Li Yajie: withlove1982@163.com

## Abbreviations

AP: Acute pancreatitis
SAP: Severe acute pancreatitis
MAP: Mild acute pancreatitis
MSAP: Moderately severe acute pancreatitis
BISAP: Bedside index of severity in acute pancreatitis
APACHE: Acute Physiology and Chronic Health Evaluation
